# Growth Performance, Gut Integrity and Intestinal Microbiome Responses of Juvenile Common Carp (*Cyprinus carpio* L.) to Probiotic and Prebiotic Supplementation

**DOI:** 10.3390/ani16030433

**Published:** 2026-01-30

**Authors:** Elshafia Ali Hamid Mohammed, Milán Fehér, Péter Bársony, Christopher Teye-Gaga, Levente Czeglédi, Csongor Freytag, Alex Váradi, Abdelhakam Esmaeil Mohamed Ahmed, Károly Pál

**Affiliations:** 1 Department of Animal Science, Institute of Animal Science, Biotechnology and Nature Conservation, Faculty of Agricultural and Food Sciences and Environmental Management, University of Debrecen, 4032 Debrecen, Hungary; feherm@agr.unideb.hu (M.F.); barsonp@agr.unideb.hu (P.B.); christopher.teye.gaga@agr.unideb.hu (C.T.-G.); czegledi@agr.unideb.hu (L.C.); 2 Doctoral School of Animal Science, University of Debrecen, 4032 Debrecen, Hungary; 3 Agricultural Research Corporation, Integrated Pest Management Research Center, Wadmadani P.O. Box 126, Sudan; 4 Department of Bioinformatics, “One Health” Institute, Faculty of Health Science, University of Debrecen, Egyetem Tér 1, 4032 Debrecen, Hungary; freytag.csongor@etk.unideb.hu; 5 Metagenomics Institute, University of Debrecen, Egyetem Tér 1, 4032 Debrecen, Hungary; varadi.alex@med.unideb.hu; 6 Institute of Food Sciences, Faculty of Agricultural and Food Sciences and Environmental Management, University of Debrecen, 4032 Debrecen, Hungary; ahmed.abdelhakam@agr.unideb.hu; 7 Doctoral School of Nutrition and Food Sciences, University of Debrecen, Böszörményi út 138, 4032 Debrecen, Hungary

**Keywords:** common carp, *Pediococcus acidilactici*, *Saccharomyces cerevisiae*, yeast extract, histomorphology, oxford nanopore sequencing, gut microbiota

## Abstract

The aim of this study was to examine the impact of the probiotics *Saccharomyces cerevisiae* (SC) and *Pediococcus acidilactici* (PA), as well as the prebiotic yeast cell wall extract (*Cyberlindnera jadinii* and *S. cerevisiae*) (YP), on the growth parameters, intestinal histomorphology, liver and gills normality, and gut microbiome of common carp (*Cyprinus carpio* L.). These feed supplements were subjected to a 60-day experimental period, during which 120 fish (26.4 ± 5.2 g) were distributed into four groups, with each group comprising 30 fish. The feed supplements were administered at a rate of 1 g/kg of body weight. Before the trial, the fish were acclimatized for two weeks, then injected with Passive Integrated Transponder (PIT) tags. The results showed that none of the feed supplements exhibited a significant effect (*p* > 0.05) on growth indices. In contrast, the villi length, villi width, muscular thickness, and crypt depth in the anterior, mid, and posterior intestine of the SC group exhibited significant (*p* < 0.05) improvements compared to the other groups. No alteration or abnormal growth were noticed in the gills and livers. The most dominant microbe genera in all groups, with abundances greater than 60% of the total, were *Cetobacterium* and *Aeromonas*. However, *Polynucleobacter*, *Nordella*, *Mycoplasma*, *Romboutsia*, and *Staphylococcus* species were present at lower abundances. The presence of *Actinobacteria* has been observed only in the intestine of fish that have been fed a diet supplemented with PA. It can be concluded that the tested probiotics and the yeast cell wall prebiotic have the potential to produce a remarkable improvement in intestinal morphology and a considerable change in the gut microbiome without notable effect on growth, livers, or gills of common carp.

## Simple Summary

Considerable attention has been gained by probiotics and prebiotics as an eco-friendly and alternative technique to antibiotics. This study investigated the effects of the probiotics *Saccharomyces cerevisiae* and *Pediococcus acidilactici*, along with the prebiotic yeast cell wall, on the growth and intestinal morphology, as well as the liver and gills, of common carp (*Cyprinus carpio* L.). The results showed a considerable improvement in intestinal morphology (e.g., villi width, muscular layer thickness) without any pathological signs on liver or gills or any change on growth parameters. In addition, considerable change in the gut microbiome was noticed when fish received diet supplemented with the tested feed supplements at 1 g/kg for 60 days in a recirculating aquaculture system.

## 1. Introduction

Rising demand for seafood and fish as an animal protein source has led to aquaculture becoming a significant part of global food production systems. Recognized as one of the fastest-growing food production sectors [1], it makes significant contributions to food security and economic development. In 2022, according to the State of World Fisheries and Aquaculture 2024, the global aquaculture production reached a record high of 130.9 million tons, highlighting aquaculture’s continued and dominant contribution to the global fish supply. The common carp was amongst the top 10 cultured species [1]. However, the occurrence of aquaculture pathogens raise a significant concern that affects both the health and growth of aquatic organisms and the economic aspects of aquaculture globally [2]. These pathogens comprise both gram-negative and gram-positive bacteria (e.g., *Streptococcus* spp.); however, gram-negative species—such as *Aeromonas salmonicida*, *Pseudomonas fluorescens*, *Vibrio harveyi*, *Yersinia ruckeri*, and *Flavobacterium psychrophilum*—are among the most common in aquaculture systems [3]. The interaction of these pathogens with environmental factors creates a detrimental effect on fish productivity, leading to substantial economic losses for farmers. Inadequate practices, such as excessive density of stocking, excessive feeding, and water pollution have been identified as contributing factors to the proliferation of pathogens in aquaculture [4,5].

The employment of antibiotics in aquaculture is a common practice, with the primary objectives of addressing disease issues and augmenting overall fish performance [6]. Nevertheless, the continual use of antibiotics in aquatic farming has generated a selective pressure on microbial ecosystems, leading to the proliferation of antibiotic-resistant bacteria capable of extensive dissemination [7]. These resistant bacteria have the potential to exert a deleterious effect on aquaculture production, consumers, and the environment [8,9,10]. Therefore, probiotics and prebiotics are considered among the most important sustainable alternatives that can eliminate the negative characteristics of antibiotics [11,12,13].

The most frequently utilized probiotics belong to the lactic acid bacteria (LAB), including genera such as *Pediococcus* [14]. Research has demonstrated that probiotics enhance the growth and health of a wide range of fish and aquatic organisms such as rainbow trout (*Oncorhynchus mykiss*), African catfish (*Clarias gariepinus*), rohu (*Labeo rohita*), Nile tilapia (*Oreochromis niloticus*), and Red seabream (*Pagrus major*) [15,16,17]. Probiotics offer their hosts a range of benefits, the most notable of which is enhanced growth performance. This phenomenon occurs through several different mechanisms, each contributing to the positive outcomes observed [18,19,20,21,22], reducing disease incidence and improving water quality, enhancing liver health [21,23,24], or enhancing the gut’s microbiota and integrity [25,26].

A variety of methods can be used to identify the bacterial composition of complex samples. Culture-based techniques have been employed to successfully identify and characterize the bacterial community of fish. However, this method is technically laborious, and the presence of non-cultivable bacteria complicates the process of microbial characterization. In this particular context, the 16S ribosomal gene has been utilized extensively for the identification of bacterial species [27]. The 16S rRNA gene is highly conserved among prokaryotes, it contains nine hypervariable regions (V1–V9) that can be used as a marker to identify bacterial species [28]. Thus, sequencing a single hypervariable region has been used to study microbial diversity in a particular environment [29,30,31]. However, sequencing different hypervariable regions can affect the estimated taxonomical diversity [32]. Furthermore, it has been demonstrated that short-read sequencing of the 16S rRNA gene cannot achieve the same taxonomic resolution as full-length sequencing (~1400 bp) [33]. In this context, the application of long-read sequencing in metagenomic studies has emerged as a novel and promising approach for examining microbial communities [34,35].

Numerous studies have investigated the use of probiotics and prebiotics in aquaculture. However, most of these studies have focused on growth performance or immune responses under challenging or stressful conditions. Little attention has been given to the effects of probiotics and prebiotics on gut integrity, liver health, and the structure of the intestinal microbiome in freshwater carp species under normal rearing conditions.

In this regard, there are few studies on the functional role of yeast cell wall–based prebiotics or probiotics, such as *Pediococcus acidilactici* or *Saccharomyces cerevisiae*, in modulating the intestinal architecture and microbial communities of juvenile common carp (*Cyprinus carpio* L.). Therefore, the aim of this study was to use Oxford Nanopore Sequencing Technology as a long-read sequencing method to identify the bacterial communities in the intestines of common carp that were fed a diet supplemented with the probiotics *Pediococcus acidilactici* or *Saccharomyces cerevisiae*, or the yeast cell wall prebiotic. The study also aimed to determine the effect of these feed supplements on the growth, intestinal morphology, and normality of the liver and gills of common carp.

## 2. Materials and Methods

### 2.1. Ethical Issues

Experiments on common carp in the current study were conducted based on ethical guidelines and legal requirements. The Department of Animal Husbandry of the Institute of Animal Science, Biotechnology and Nature Conservation at the University of Debrecen obtained the necessary permit for these experiments from the Animal Welfare Committee of the University of Debrecen (15/2019/DE MÁB Kovács László). This permit, issued by the Hajdú-Bihar County Government Office on 5 March 2020, has a validity of five years (until 5 March 2025) and was authorized by Dr. Sándor Mihálka, Chief Veterinary Inspector.

### 2.2. Feed Composition

The commercially available fish feed used in this study was Aqua Start (Garant Aqua, Pöchlarn, Austria), an extruded diet provided as 1.5 mm pellets and formulated to support optimal growth and health of juvenile fish. According to the manufacturer, the feed composition on a dry matter basis consisted of 52.0% crude protein, 18.0% crude fat, 0.5% crude fiber, 9.5% crude ash, 1.5% phosphorus, and 1.8% calcium. The diet was additionally fortified with essential vitamins and minerals, including vitamin A (15,000 IU/kg), vitamin D_3_ (2000 IU/kg), vitamin E (200 mg/kg), and stabilized vitamin C (250 mg/kg). The major ingredients listed by the manufacturer included wheat, fish meal, corn gluten, soybean protein concentrate, fish oil, and a vitamin–mineral premix. This feed served as the basal diet for all experimental groups.

### 2.3. Fish Preparation and PIT Tagging

**Figure 1:**
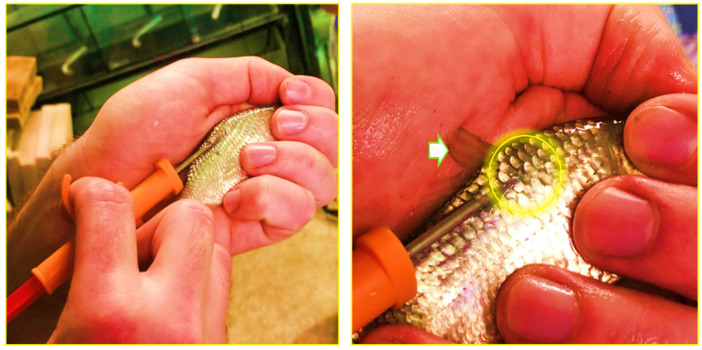
The injection of a Passive Integrated Transponder (PIT) tag to each fish for the purpose of individual identification. The white arrow shows the dorsal fin, and the yellow circle indicates the tagging site, which is located below the base of the dorsal fin.

Common carp (26.4 ± 5.2 g) were reared in a recirculating system at the Fish Laboratory of the University of Debrecen, Hungary. The carp strain was “Hajdúszoboszló scaly”, and the artificial propagation was made by researchers of the Department of Animal Husbandry with the local fish farmer’s stock. During the propagation one batch of fish eggs was used and mixed with two different semen from two melters for the better fertilization (the standard procedure during the artificial propagation). After the acclimatization for 14 days, 120 fish were randomly distributed into 12 fish tanks and 4 treatments (10 fish per tank, 30 fish per treatment); the system was equipped with mechanical and aerated biofilter. Fish tanks were circular with a diameter of 1.44 m and a water depth of 0.75 m. The water capacity of each tank was 350 L. Before the distribution in fish tanks, individuals were anesthetized using clove oil [36] then assigned to passive integrated transponder (PIT) tags (HUN-CHIP 2, 12 × 12 mm FDX-B Standard chip sterilized with EO gas) to monitor fish performance during the experiment. Before and after the chip was placed under the skin on the right side of the back, a disinfection with Betadine was made. Briefly, all fish were weighed prior to the experiment (g) and then individually injected with the PIT tag in front of the dorsal fin (Figure 1). After injection, tag function was checked using a hand-held PIT tag reader (iD Porte Ltd.; Guernsey, Channel Islands). The tagged fish were then held in the fish tanks for 72 h to recover from PIT injection stress before being assigned to the experimental diets for 60 days.

### 2.4. The Experimental Diets and Animal Housing

Three different feed additives, each produced by the Lallemand Animal Nutrition (Montreal, QC, Canada), were tested: Bactocell^®^ is based on the lactic acid bacterium *Pediococcus acidilactici* CNCM I-4622 MA 18/5M, containing ~1 × 10^10^ CFU/g. Levucell^®^ contains the yeast strain *Saccharomyces cerevisiae* var. *Boulardii* CNCM I-1079 in ~1 × 10^9^ CFU/g concentration, according to the manufacturer’s specifications. YANG^®^ is a commercial product from the yeast cell wall extract of *Cyberlindnera jadinii* and *S. cerevisiae.* Living cell numbers were checked by plating after the experiment and confirmed the expected germ number. The experimental diets were prepared by mixing the commercial feed pellets (Section 2.2) with the feed additives and sunflower oil (20 mL/kg), following the protocols provided by the product manufacturers. The control diet was treated identically, except that no additives were included. All diets were subsequently dried at 39 °C for 24 h. After that, it was left at room temperature for daily use.

The feeding trial was conducted with 3 replicates (3 tanks/treatment) and 4 groups as follows: fish in the control group received a commercial feed only (C), whereas animals in the other 3 groups received diet supplemented with 1 g/kg of *P. acidilactici* (PA), *S. cerevisiae* (SC), and YANG^®^ (YP), respectively. The RAS system was equipped with a LINN PR5A—Profi-Automatic Feeder (LINN Gerätebau GmbH, Lennestadt-Oberelspe, Germany), programmed feed three times a day (08:00, 14:00, and 20:00) with feeding rates of 3% of the total biomass. The total biomass was recalculated every two weeks based on body weight measurements of tagged fish, and the daily feed ration was adjusted accordingly. The lighting conditions were 12 h of light and 12 h of darkness. The removal of feces and uneaten feed was regular practice, performed as needed.

### 2.5. Water Quality

Throughout the experimental period, the dissolved oxygen concentration (mg/L) was kept at 6.78 ± 0.3, the temperature (°C) at 25.9 ± 0.9, and the pH at 7.4 ± 0.14. Meanwhile, the concentrations of nitrate (NO_3_^−^), nitrite (NO_2_^−^), and ammonium (NH_4_^+^) were monitored on a regular basis using a DR3900 Spectrophotometer (Hach, Loveland, CO, USA). The recorded values were within the optimal range: TDS concentration was 443 ± 6.7 ppm; nitrate concentration was 5.48 ± 2.97 mg/L; nitrite concentration was 0.24 ± 0.3 mg/L; and ammonium concentration was 0.37 ± 0.18 mg/L.

### 2.6. Growth Indices Measurements

Every two weeks, all the fish from all the tanks were collected. The wet body weight (BW, g) was measured. Feed conversion ratio (FCR, g/g) was calculated using the total amount of feed offered during each two weeks, as uneaten feed was not collected or quantified. Survival rates (S%); and weight gain (WG, g); were calculated based on the following mathematical formulas:

(1) S (%) = (harvested individuals/stocked individuals) × 100
(2) WG (g) = Wf − Wi
(3) FCR (g/g) = F/(Wf − Wi)

where Wf is the final body weight (g), Wi is the initial wet body weight (g), and F is feed intake (g) (F was calculated based on the average of biomass).

Fish were fasted for 24 h prior to weighing to reduce the effect of gut contents on body weight.

### 2.7. Histological Examinations

After the feeding experiment ended (60 days), three carps from each group were selected randomly and anesthetized by bathing for 5 min in a clove oil solution at 0.025 mL/L at 23 °C [36]. The fish intestine, liver, and gills were carefully withdrawn. The anterior, posterior, and mid-intestine of each fish were clearly identified. The intestinal and liver samples were then washed in phosphate-buffered saline (PBS) solution and fixed in 10% buffered formalin until analysis. Histochemical investigation was carried out as follows: samples were dehydrated in ethanol, cleaned, and embedded in paraffin. Subsequently, the fixed tissues were sectioned at a thickness of 5 µm using a rotary microtome (Leica 2025, Wetzlar, Germany). To examine the intestinal microstructure, liver and gills normality, hematoxylin-eosin (H&E) staining was conducted on 12 samples per group (*n* = 3 fish per group, 4 cross sections per individual). A light microscope (BX61, Olympus, Tokyo, Japan) connected with camera (DP71, Olympus, Tokyo, Japan) was used to capture images of stained cross sections [37]. The slides were observed under a light microscope with a magnification of 40× for the intestine and 100× for liver and gills. Histological analysis was performed using 3 fish per treatment as biological replicates. From each fish, 3 intestinal cross-sections were considered as technical replicates. Only intact villi without damage were considered for analysis and their orientation was checked carefully. The length of villi (LV), villus width (VW), crypt depth (CD), and the muscular layer thickness (ML) were measured (μm). Measurements were conducted with Olympus CellSens Entry Software (Olympus Corporation, Tokyo, Japan, version 1.16).

### 2.8. Analysis of Microbial Communities of the Intestine

#### 2.8.1. DNA Extraction

Genomic DNA was extracted using the Zymo Research DNA extraction kit (D4300) (Zymo Research Corporation, Irvine, CA, USA), following the manufacturer’s instructions. DNA was eluted in 60 µL of ultrapure DNase/RNase-free distilled water (Invitrogen, 10977-035). DNA integrity was assessed by 1% agarose gel electrophoresis, and DNA concentration was quantified according to the manufacturer’s protocol using the Qubit™ dsDNA HS Assay Kit (Thermo Fisher Scientific, Waltham, MA, USA).

#### 2.8.2. Library Preparation

Library preparation was performed using the 16S Barcoding Kit 24 V14 (SQK-16S114.24; Oxford Nanopore Technologies, Oxford, UK) according to the manufacturer’s instructions. The full-length 16S rRNA gene was amplified using the barcoded primer pair 27F and 1492R provided in the kit. PCR amplification was carried out using LongAmp™ Hot Start Taq 2X Master Mix (NEB, M0533S) on a VWR Uno 96 thermal cycler. Thermal cycling conditions consisted of an initial denaturation at 95 °C for 1 min, followed by 25 cycles of denaturation at 95 °C for 20 s, annealing at 55 °C for 30 s, and extension at 65 °C for 2 min, with a final extension at 65 °C for 5 min. PCR products were held at 4 °C until further processing.

#### 2.8.3. Bioinformatic Analysis

Basecalling of raw sequencing data was performed using Guppy with the Super High Accuracy v4.3.0 model (Oxford Nanopore Technologies, Oxford, UK). Reads were quality filtered to retain sequences with a minimum Q-score of 10. High-quality reads were subsequently demultiplexed and trimmed, retaining sequences with a length between 1200 and 1700 bp. Taxonomic classification was performed using EMU version 3.4.5, specifically optimized for full-length 16S rRNA gene analysis. Species represented by fewer than ten reads were excluded from the final dataset. Samples with fewer than 5000 total reads after filtering were excluded from downstream analyses.

Alpha diversity was estimated using the Shannon and Simpson diversity indices, while evenness was calculated as the ratio of Shannon diversity to the natural logarithm of species richness (Pielou’s evenness).

### 2.9. Statistical Analysis

The obtained data was analyzed using IBM SPSS Statistics for Windows, Version 24.0 (IBM Corp., Armonk, NY, USA). One-way analysis of variance (ANOVA) was carried out, the variance equality was checked by Bartlett’s test and LSD was used for the post hoc tests. Before the analysis, the data were also checked for normality by using the Shapiro–Wilk test. Differences in alpha diversity between the four treatment groups were tested using the Kruskal–Walli’s test. Data were presented as means, and the significance level for differences was set at *p* < 0.05. Differences among groups were considered significant at *p* < 0.05. Then the results were expressed as mean ± std (standard deviation of the mean).

## 3. Results

### 3.1. Growth Performance

The results showed that fish in all treatment groups exhibited progressive increases in body weight (BW, g) and weight gain (WG, g) from 17 November 2023 to 18 January 2024 (Table 1), with the highest final BW recorded in the PA supplemented diet (106.8 ± 25.8 g), followed by YP (105.9 ± 25.3 g), the control (103.8 ± 30.8 g), and SC (99.9 ± 25.5). Similarly, the highest WG was recorded in the YP (34.34 ± 16.2 g) and PA (36.1 ± 20.7 g) groups. Feed conversion ratio (FCR) improved over time and was similar across all groups at the end of the trial (1.0 ± 0.1), while survival rate (S%) remained at 100% in all groups throughout the study. There were no significant differences (*p* > 0.05) among the control and supplemented groups in terms of BW, WG, FCR, or S% at any sampling point.

### 3.2. The Intestinal Histoarchitecture

**Figure 2:**
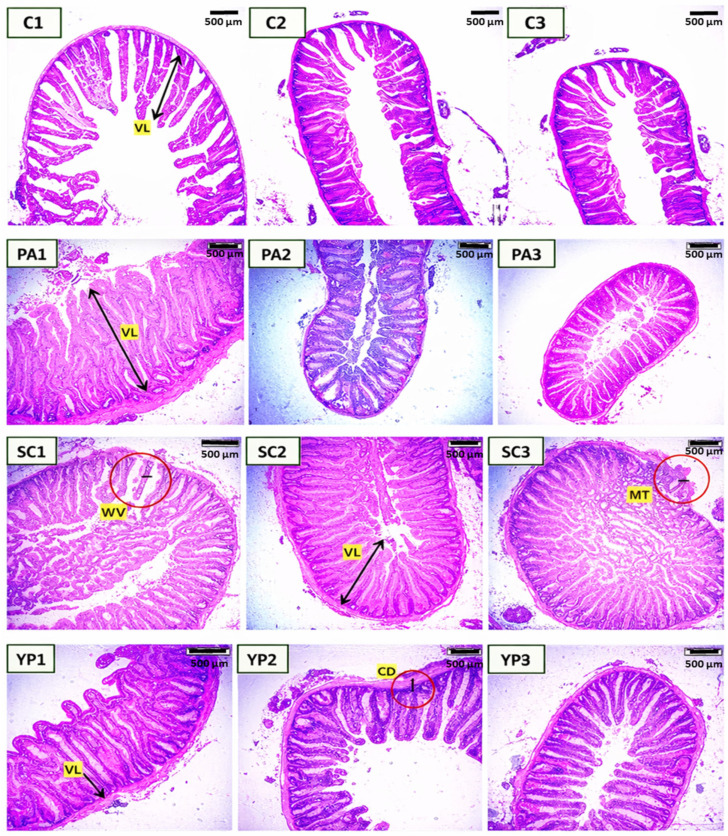
Histological cross sections (H&E) of anterior (1), mid (2), and posterior intestine (3) of common carp showed the effect of *P. acidilactici* (PA), *S. cerevisiae* (SC), and the yeast cell wall prebiotic (YP), in comparison with the control (C). VL: villus length, MT: muscular layer thickness, VW: villi width, CD: crypt depth. Bar = 500 μm. Magnification = 100×.

Dietary treatments significantly influenced the intestinal architecture of the anterior, mid, and posterior segments of *C. carpio* after 8 weeks (Table 2, Figure 2). In the anterior intestine, villi length, width, and muscular thickness were significantly higher (*p* < 0.05) compared to the untreated control. The probiotic PA exhibited the highest villi length in the anterior intestine (1915.8 ± 398.2 µm) followed by SC (1637.4 ± 227.3 µm), YP (1452.2 ± 312.4 µm), and the control (973.1 ± 199.3 µm), respectively.

In the mid-intestine (Table 3), villi length and width significantly improved (*p* < 0.05) in fish that received diet supplemented with *S. cerevisiae*, which had the longest villi (1350.20 ± 82.0 µm) and widest villi (176.9 ± 49.7 µm); however, no significant differences (*p* > 0.05) were observed among the other groups. Crypt depth and muscular thickness did not differ significantly in the mid-intestine, though numerical changes were observed in supplemented groups.

Regarding the posterior intestine, dietary supplementation with SC had positive effects on intestinal morphology of *C. carpio* (Table 4). Villus length differed significantly between groups (*p* = 0.015). The highest villus length was observed in the SC group (1167.28 ± 505.9 µm), followed by YP (847.68 ± 282.7 µm), and PA (784.4 ± 171.2 µm). The control group had the shortest villi (779.34 ± 188.9 µm). Villus width, muscular thickness, and crypt depth did not differ significantly among treatments (*p* > 0.05), but all supplemented groups had higher values than the control group. These results suggest that dietary probiotics and prebiotics improve posterior intestinal structure by enhancing villus length without negatively affecting other histomorphological parameters.

### 3.3. Gills and Liver Histology

**Figure 3:**
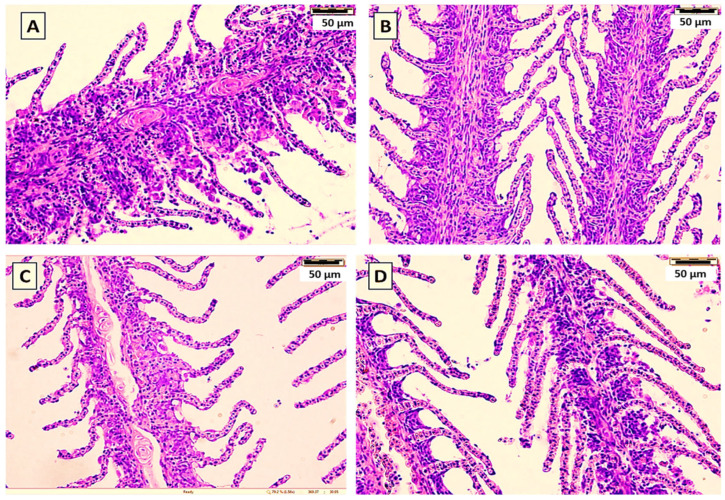
Histological sections (H&E) of the gills of common carp showed the effect of the yeast cell wall (**A**), *S. cerevisiae* (**B**), *P. acidilactici* (**C**) in comparison with the control (**D**) for 60-day feeding trial. Bar = 50 μm. Magnification = 100×.

**Figure 4:**
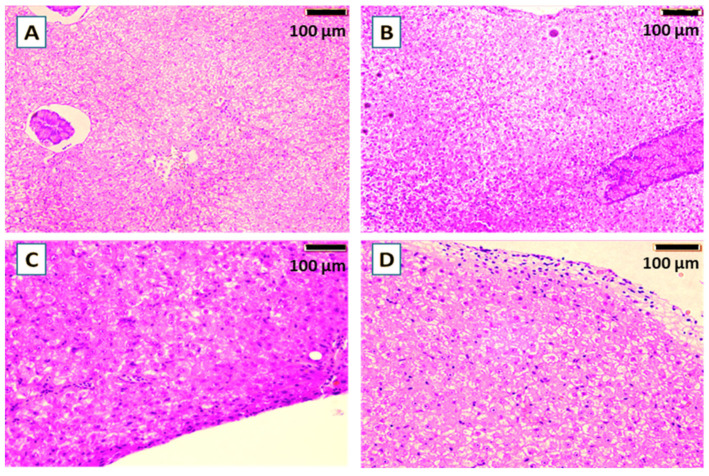
Histological sections (H&E) of the liver of common carp showed the effect of the yeast cell wall (**A**), *S. cerevisiae* (**B**), *P. acidilactici* (**C**) in comparison with the control (**D**) for 60-days trial. Bar = 100 μm. Magnification = 100×.

Histological examination of common carp gills after the 60-day feeding trial revealed no differences between groups supplemented with yeast cell wall prebiotic, the probiotics *S. cerevisiae* or *P. acidilactici*, or the control feed (Figure 3). Gill lamellae and filaments in all groups exhibited normal architecture, with unbroken epithelial lining and no hyperplasia, fusion, or necrosis. The secondary lamellae were also clearly defined. Similarly, histological examination of liver (Figure 4) revealed no visible pathological changes in any of the treatment groups. Normal liver architecture, characterized by healthy hepatocytes, suggests that none of the tested feed additives had any negative effects on liver morphology.

### 3.4. Alteration in the Intestinal Microbiota

**Figure 5:**
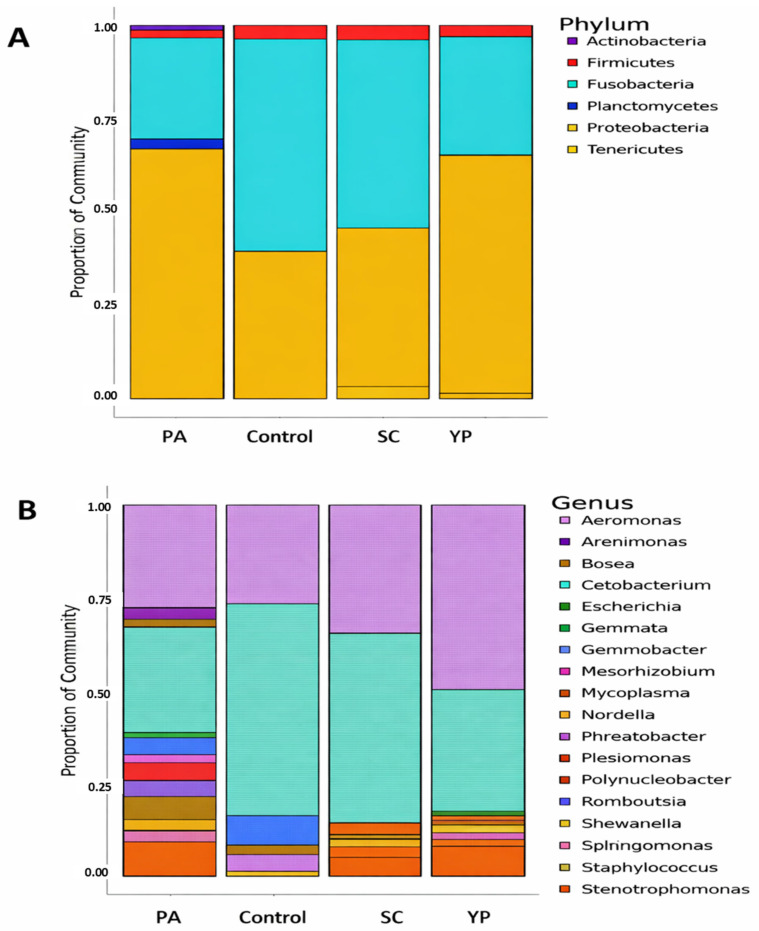
The relative abundance of bacterial phyla (**A**) and genera (**B**) in the intestine of *C. carpio* was assessed after 8 weeks of receiving a diet supplemented with PA, SC, YP, or a control diet only. This assessment was conducted using 16S rRNA gene sequencing. “PA”: *Pediococcus acidilactici*, “SC”: *Saccharomyces cerevisiae*, “YP”: Yeast prebiotic.

Across all dietary supplements and the control, the microbial community is predominantly composed of *Proteobacteria* and *Fusobacteria (*Figure 5), though their proportions vary notably between groups. PA exhibited a high abundance of *Proteobacteria* (~65%), followed by *Fusobacteria* (~26%), while the control and SC were dominated by *Fusobacteria* (>55%), with reduced proportions of *Proteobacteria* (<43%). In addition, YP group showed a profile like PA, with *Proteobacteria* again comprising most of the bacterial community. Minor phyla including *Firmicutes*, *Actinobacteria*, *Planctomycetes*, and *Tenericutes* are present in low proportions across all groups, with *Firmicutes* being slightly more prominent in samples PA and YP. Notably, *Planctomycetes* appear only in PA group.

The genus-level bacterial composition across the four groups (PA, SC, YP, and the control), revealed a considerable variation in microbial community. In group PA, the bacterial community is primarily dominated by *Cetobacterium*, which accounts for approximately 26.5% of the total relative abundance (~0.265), closely followed by *Aeromonas* at around 26.2% (~0.262). *Stenotrophomonas* is the third most abundant genus, contributing to about 8.6% (~0.086), while *Polynucleobacter* and *Nordella* represent approximately 5.9% (~0.0593) and 4.6% (~0.046), respectively. Other genera, such as *Mycoplasma*, *Romboutsia*, and *Staphylococcus*, are present at lower abundances, each contributing less than 4% (~0.04) to the community structure.

In group SC, the bacterial community is predominantly composed of two genera: *Cetobacterium* and *Aeromonas*, which together account for nearly 85% of the total relative abundance. *Cetobacterium* is the most abundant genus, representing 50.5% (~0.505) of the community, followed by *Aeromonas* at 34.1% (~0.341). *Stenotrophomonas* contributes 4.9% (~0.049), while *Mycoplasma* is present at 3.1% (~0.031). All remaining genres constitute less than 3% of the community. This composition indicates a low-diversity microbial profile heavily dominated by *Cetobacterium*, with moderate contributions from *Aeromonas* and minimal representation of other taxa, suggesting a highly specialized or selective microbial environment in group SC.

In group YP, the bacterial community is heavily dominated by *Aeromonas*, which comprises approximately 48.4% of the total abundance (~0.484), followed by *Cetobacterium* at around 31.6% (value = 0.316). *Stenotrophomonas* is the third most abundant genus, contributing about 7.6% (~0.076), while each of the remaining genera—such as *Polynucleobacter*, *Mycoplasma*, and *Staphylococcus*—contributes less than 2% (<0.02).

In group C, the bacterial community is overwhelmingly dominated by *Cetobacterium*, which constitutes approximately 56.2% of the total abundance (~0.562), indicating a strong single-genus dominance. This is followed by *Aeromonas* at 26.6% (~0.266), while *Gemmobacter* and *Romboutsia* contribute approximately 8.0% (~0.08) and 4.4% (~0.044), respectively. Other genres, including *Plesiomonas* and *Shewanella*, are present at lower levels, each accounting for less than 4% of the community (~0.04). This composition suggests a moderately diverse bacterial structure with a pronounced dominance by *Cetobacterium*, potentially reflecting specific environmental or host-associated factors shaping the microbiota in group C.

**Figure 6:**
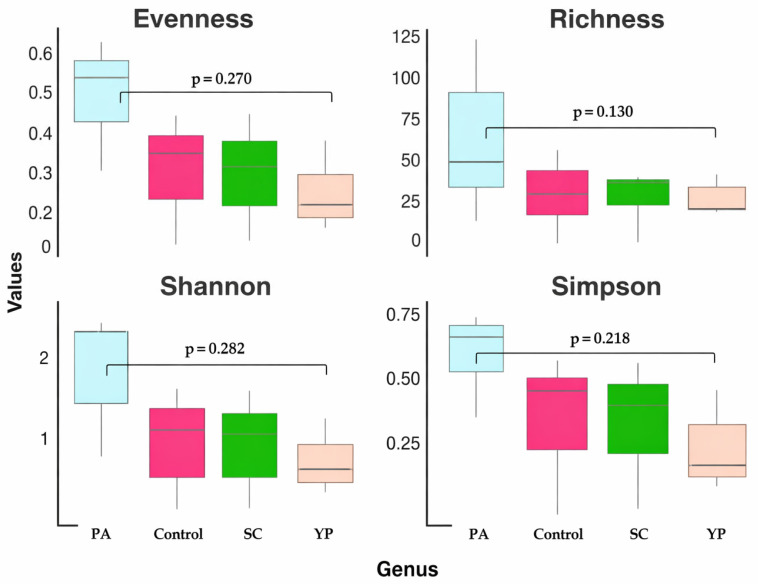
Boxplots showing four alpha diversity metrics (evenness, richness, Shannon index, and Simpson index) at the genus level for the bacterial communities in the intestines of *C. carpio* that received a prebiotic- and probiotic-supplemented diet for 8 weeks.

The alpha diversity metrics, including evenness, richness, Shannon, and Simpson indices, across the four groups (PA, SC, YP, and the control group) at the genus level are shown in Figure 6. In PA, the highest metric across all feed supplements were recorded, including the highest evenness (~0.55–0.60), richness (~60–70), Shannon index (~2.2), and Simpson index (~0.75). In contrast, group YP shows the lowest diversity, with notably reduced evenness (~0.2), richness (~25), Shannon (~1.0), and Simpson (~0.35) indices, reflecting a community dominated by a few genera. Regarding the control and SC groups, intermediate diversity levels with similar evenness (~0.35–0.40), richness (~30–40), and diversity indices (Shannon ~1.2–1.4 and Simpson ~0.55–0.60) were recorded. Overall, while numerical differences in alpha diversity were evident among treatments, the *p*-values indicate that these variations were not statistically significant.

## 4. Discussion

With the rapidly growing global population, sustainable aquaculture practices are essential for providing high-quality protein [38]. Probiotics and prebiotics have recently gained popularity in the aquaculture industry as effective methods of improving fish health [38,39,40]. Generally, extensive studies have been conducted to investigate the effects of probiotics as a promising technique in fish farming [41,42,43,44,45,46]. In our research, feed supplements containing probiotics and prebiotics for common carp produced promising results that could serve as a basis for further research into enhancing growth, gut integrity, and intestinal microbiota. Growth parameters are crucial in aquaculture as they indicate health and productivity of fish, and can be affected by factors such as the quality of the feed and environmental conditions [47]. LAB-bacteria such as *P. acidilactici* were noticed to improve growth, survival (%), feed digestion, and the immune system of different aquatic animals [40,41]. To the best of our knowledge, only a few studies have specifically examined the effects of *S. cerevisiae* and *P. acidilactici*, as well as the yeast cell wall-based prebiotic, on the growth indices, morphometric features of the intestine, and gut microbiota of cultured carp. Furthermore, there are no published studies that have examined the effects of yeast cell wall prebiotics on cultured common carp.

### 4.1. Growth Performance

Previous studies have reported growth-promoting effects of probiotics and prebiotics in fish; however, these effects are strongly influenced by species, probiotic strain, inclusion level, and experimental conditions. Growth enhancement following dietary supplementation with *Saccharomyces cerevisiae* or *Pediococcus acidilactici* has been reported in species such as Nile tilapia, sea bass, and striped catfish (*P. hypophthalmus*) [48,49,50].

In our study, dietary supplementation with *S. cerevisiae* or *P. acidilactici*, or the yeast cell wall prebiotic did not result in significant changes in body weight, feed conversion ratio, or survival compared with the control diet that could indicate that the tested feed additives were as safe as the control diet under the given experimental conditions. These results indicate that the tested feed supplements had no effect on growth performance under the current experimental conditions, confirming their safety when included in the diet. Our results are somewhat consistent with those of Hoseini et al. [51] and Standen et al. [52], who found that *S. cerevisiae* and *P. acidilactici* have no effect on the growth of rainbow trout and Nile tilapia.

### 4.2. The Intestinal Histoarchitecture, Liver, and Gills

In the present study, the probiotic *P. acidilactici* showed significant improvements in the intestinal architecture, especially the anterior intestine of the common carp, including villi length and width, muscular thickness, and crypt depth (*p* < 0.05). These findings are consistent with Eissa et al. [24], who reported that the feed supplementation with *P. acidilactici* can positively enhance the development of internal organs, such as length of intestinal villi and liver of Nile tilapia (*O. niloticus*). Additionally, our results are also somewhat consistent with those of Ashouri et al. [53], Wang et al. [54], and Zheng et al. [55], who detected improvement in villus surface, the width of villi, and crypt depth when lactic acid probiotics were used as feed supplement. Considering the morphological parameters of intestinal villi, it is evident that they are directly correlated with nutrients absorption and digestion efficiency [56].

In this study, *S. cerevisiae* also considerably improved the width and length of the intestinal villi of common carp compared to the control group. The efficacy of this probiotic was also been reported by Boonanuntanasarn et al. [50], who noticed that dietary supplementation with *S. cerevisiae* (1 × 10^8^ CFU) remarkably improved villus length (μm) in the anterior and mid-intestines of catfish (*Pangasianodon hypophthalmus*). In addition, the efficacy of *S. cerevisiae* for improving intestinal architecture has been confirmed in sea bream (*Sparus aurata*) [57] and Atlantic salmon (*Salmo salar*) [58]. Increased villi length and width indicate an enlarged absorptive area of intestinal tissue, which improves growth, development, and feed utilization. However, the precise mechanisms through which probiotics and prebiotics enhance intestinal absorption are not fully understood. The cells at the tip of the villi are continuously sloughed off, and the intestinal epithelium’s renewal rate is high enough to replace them [59]. Additionally, *S*. *cerevisiae*’s beneficial effect on intestinal histomorphology could be attributed to the monosaccharides that compose its cell wall. These monosaccharides have been shown to positively impact the intestinal villi and microvilli of various fish species [59]. Furthermore, several studies have shown that yeast-based feed supplements enhance the intestinal indices of Nile tilapia [60] and Jian carp (*C. carpio* var. Jian) [61]. These region-specific effects may be related to differences in the dietary supplements used. For example, Yassine et al. [62] reported that supplementing the diet with *Lactobacillus plantarum* enhances the length and width of the intestine villi of common carp. Generally, our findings suggest that dietary probiotics and prebiotics improve posterior intestinal structure by enhancing villus length without negatively affecting other histomorphological parameters.

In gills histology, the lamellae in all groups exhibited normal architecture, with an unbroken epithelial lining and no hyperplasia, fusion, or necrosis. These observations suggest that including probiotic and yeast-based supplements in the diet did not have any histopathological effects on gill tissue. Maintenance of normal gill structure suggests that the tested additives were non-toxic and well tolerated by common carp under experimental conditions.

In liver histology, liver sections of common carp revealed no visible pathological changes in any of the treatment groups. Normal liver architecture, characterized by healthy hepatocytes, suggests that none of the tested feed additives had any negative effects on liver morphology.

There have been similar reports that probiotic feed additives have no harmful impact on the gills and liver morphology of fish [24]. These results provide further support for the safety and suitability of these additives in aquaculture.

### 4.3. The Intestinal Microbiota

Unlike traditional culture-dependent methods, which are limited to detecting only cultivable microorganisms and often overlook most gut-residing bacteria, Oxford Nanopore sequencing technology provides a comprehensive, high-resolution profile of both abundant and rare microbial taxa. The intestine of any aquatic animal provides an ideal environment for beneficial microbes to colonize and grow [63]. The composition of the intestinal microbiome can influence host physiology, growth, and health [46]. A diversity of intestinal microbes is generally considered beneficial for the health of the host. Therefore, an investigation was conducted to examine the impacts of probiotics, specifically *S*. *cerevisiae*, and *P. acidilactici* as well as yeast prebiotics, on the gut microbiome of *C. carpio*.

In our results, *Actinobacteria* was detected only with low abundance (~0.011) in the intestine of fish which received a diet supplemented with *P. acidilactici*. *Actinobacteria*, especially *Streptomyces* spp., have garnered interest as potential probiotics in aquaculture due to their numerous advantages, including their capacity to synthesize bioactive compounds and their role in nutrient cycling. Their contribution to fish health and growth performance are significant, making them notable players in sustainable aquaculture practices [64,65]. For example, Arghideh et al. [66] reported that *Streptomyces chartreusis* (KU324443) enhanced the growth parameters, antioxidant activity, and serum immune parameters in common carp (*C. carpio*) fingerlings. In addition, Das et al. [67] found positive impacts on growth performance of black tiger shrimp fed diets supplemented with *Streptomyces* spp. probiotic. Furthermore, the beneficial effect has been noticed on shrimp by García-Bernal et al. [68] and AftabUddin et al. [69].

In our study, all groups recorded a high abundance of the *Cetobacterium* genus (26–50%). The *Cetobacterium*, which belongs to the *Fusobacteriota* phylum is known to be associated with polypeptide and protein digestion, benefits the host as a good source of vitamin B_12_, butyrate, and antibacterial compounds [70]. The *Cetobacterium* genus is found in a range of freshwater fish species [71]. Accordingly, this genus is considered an indicator of healthy fish [72,73]. For example, Ma et al. [74] observed that the relative abundance of *Cetobacterium* decreased from 23% in healthy intestines to 0.5% in diseased intestines of Yunlong grouper (*Epinephelus moara *× *Epinephelus lanceolatus*).

*Aeromonas* was the second largest genius in all groups but is generally recognized as a pathogen and commonly found in aquatic environments. *Aeromonas* spp. has also been recognized as a causative agent of human and animal diseases, including septicemia, which is characterized by the presence of *A. septicemia* [75]. *Aeromonas* spp. has been identified as a significant bacterial contaminant that can compromise the integrity of aquatic ecosystems and organisms [76]. However, it is also constituent of the fish microbiome, which plays a substantial role for the fermentation of organic substances, the expression of antibacterial activity, and the degradation of cellulose [77]. Interestingly, despite the high relative abundance of *Aeromonas* in YP and other groups, all fish exhibited 100% survival rates, normal intestinal histology, and typical growth performance. This suggests that the detected *Aeromonas* sequences likely represent non-pathogenic species rather than known pathogens, such as *A. hydrophila* and *A*. *salmonicida*. The genus *Aeromonas* includes both pathogenic and non-pathogenic species, and in the context of this study, the high abundance likely reflects a normal gut microbial population rather than disease infection.

The analysis of alpha diversity provided insight into the diversity of microbes within the sample; however, the absence of beta diversity analysis is a limiting factor of this study. Future studies with an increased sample size and greater sequencing depth are recommended to enable comprehensive comparisons of beta diversity.

## 5. Conclusions

In response to the increasing demand for seafood, aquaculture has become a key contributor to global food production; however, disease outbreaks and management challenges continue to limit productivity. The widespread use of antibiotics raises concerns regarding antimicrobial resistance, highlighting the need for sustainable alternatives. Based on the findings of the present study, dietary supplementation with probiotics and prebiotics can be considered a safe approach in aquaculture. Considering these, probiotics and prebiotics are being explored as sustainable alternatives to antibiotics.

Neither the probiotics *S. cerevisiae* and *P. acidilactici* and the yeast cell wall extract at a concentration of 1 g/kg for an 8-week trial did affect growth indices of common carp. However, all the tested feed supplements resulted in a significant improvement in the anterior intestine including villi length and width, muscular thickness, and the crypt depth. In addition, the yeast probiotic (*Saccharomyces cerevisiae*) exhibited a significant improvement of villi length in all parts of the intestinal tissue of *C. carpio* (anterior, mid, and posterior intestine). Nevertheless, other supplements had no significant effect on mid-intestines and posterior intestine of *C. carpio.*

The genus-level bacterial composition across the four groups revealed a considerable variation in microbial community. In *P. acidilactici* group, the bacterial community is dominated by *Cetobacterium*, *Aeromonas*, *Stenotrophomonas*, while *Polynucleobacter*, *Nordella*, *Mycoplasma*, *Romboutsia*, and *Staphylococcus* are present at lower abundances, each contributing less than 9% (~0.09) to the community structure. In *S. cerevisiae* supplemented diet, the bacterial community is also predominantly composed of *Cetobacterium* and *Aeromonas*, which together account for nearly 85% of the total relative abundance. In the yeast cell wall prebiotic, the bacterial community is heavily dominated by *Aeromonas*, which comprises approximately 48.4% of the total abundance (~0.484), followed by *Cetobacterium* and *Stenotrophomonas*. However, *Actinobacteria* was detected only with low abundance (~0.011) in the intestine of fish receiving a diet supplemented with *P. acidilactici*.

## Figures and Tables

**Table 1:** Growth parameters of common carp (*Cyprinus carpio*) receiving different dietary supplements over an 8-week trial.

Growth Parameters	Feed Supplements	17 November 2023	4 December 2023	18 December 2023	2 January 2024	18 January 2024
	Control	26.3 ± 5.2	36.2 ± 9	51.5 ± 12	71.9 ± 18	103.8 ± 30.8
	PA	26.6 ± 4.5	36.8 ± 7.7	50.7 ± 10.7	71.7 ± 14	106.8 ± 25.8
BW (g)	SC	27 ± 5.6	35.2 ± 8.3	50.5 ± 12.9	69.4 ± 17	99.9 ± 25.5
	YP	26.4 ± 5.2	35.9 ± 8.6	51.1 ± 10.3	71.6 ± 16	105.9 ± 25.3
	*p*-value	0.886	0.89	0.95	0.92	0.75
	Control	-	9.93 ± 5.6	14.94 ± 6.3	21.11 ± 15.5	31.86 ± 19.8
	PA	-	10.21 ± 7.6	11.54 ± 11.1	20.01 ± 10.7	36.1 ± 20.7
WG (g)	SC	-	8.2 ± 4.2	15.37 ± 5.9	18.89 ± 4.6	30.4 ± 14.4
	YP	-	9.94 ± 5.4	16.2 ± 6.6	19.53 ± 7.7	34.34 ± 16.2
	*p*-value		0.53	0.07	0.85	0.62
	Control	-	1.9 ± 0.4	1.4 ± 0.1	0.9 ± 0.1	1.0 ± 0.2
	PA	-	2.0 ± 0.7	1.6 ± 0.4	0.9 ± 0.1	1.0 ± 0.2
FCR (g/g)	SC	-	2.1 ± 0.8	1.4 ± 0.2	1.0 ± 0.01	1.0 ± 0.1
	YP	-	2.1 ± 0.5	1.3 ± 0.05	0.9 ± 0.1	1.0 ± 0.1
	*p*-value		0.97	0.54	0.71	0.9
	Control	-	100 ± 0	100 ± 0	100 ± 0	100 ± 0
	PA	-	100 ± 0	100 ± 0	100 ± 0	100 ± 0
S%	SC	-	100 ± 0	100 ± 0	100 ± 0	100 ± 0
	YP	-	100 ± 0	100 ± 0	100 ± 0	100 ± 0

“PA”: *Pediococcus acidilactici*, “SC”: *Saccharomyces cerevisiae*, “YP”: Yeast prebiotic. The data presented as mean ± SD.

**Table 2:** Effects of the tested feed supplements on the anterior intestine of *C. carpio* (Mean ± SD).

Parameters (µm)	Control	PA	SC	YP	*p*-Value
Villi length	973.1 ± 199.3 a	1915.8 ± 398.2 bc	1637.4 ± 227.3 c	1452.2 ± 312.4 b	0.000
Villi width	159.79 ± 34.8 a	189.1 ± 24.5 c	130.2 ± 14.7 b	143.2 ± 23.7 b	0.000
Muscular thickness	65.2 ± 20.4 a	101.2 ± 34 ab	103.1 ± 22.0 a	145.4 ± 84.2 b	0.007
Crypt depth	39.5 ± 7.5 a	66.3 ± 11.8 a	74.7 ± 32.0 a	103.3 ± 98.7 a	0.074

Different letters within rows indicate significant differences (LSD, *p* < 0.05). “PA”: *Pediococcus acidilactici*, “SC”: *Saccharomyces cerevisiae*, “YP”: Yeast prebiotic.

**Table 3:** Effects of the tested feed supplements on the mid-intestines of *C. carpio* (Mean ± SD).

Parameters (µm)	Control	PA	SC	YP	*p*-Value
Villi length	1042 ± 175 a	1069 ± 300 a	1350 ± 202 b	1132 ± 182 a	0.015
Villi width	133.1 ± 20.5 a	110.7 ± 22.1 a	176.9 ± 49.7 b	139.1 ± 43.5 a	0.003
Muscular thickness	77.9 ± 27.8 a	69.2 ± 24.7 a	93.6 ± 32.4 a	96.4 ± 25.1 a	0.107
Crypt depth	46.8 ± 10.6 a	43.8 ± 16.2 a	48.0 ± 6.0 a	52.1 ± 13.9 a	0.509

Different letters within rows indicate significant differences (LSD, *p* < 0.05). “PA”: *Pediococcus acidilactici*, “SC”: *Saccharomyces cerevisiae*, “YP”: Yeast prebiotic.

**Table 4:** Effects of the tested feed supplements on the posterior intestine of *C. carpio* (Mean ± SD).

Parameters (µm)	Control	PA	SC	YP	*p*-Value
Villi length	779.3 ± 188.7 a	784.4 ± 171.2 a	1167.2 ± 505.9 b	847.6 ± 282.7 a	0.028
Villi width	114.8 ± 18.2 a	116.0 ± 25.5 a	116.0 ± 58.1 a	116.0 ± 57.7 a	0.111
Muscular thickness	67.0 ± 26.1 a	71.1 ± 16.1 a	78.0 ± 18.5 a	74.2 ± 23.4 a	0.722
Crypt depth	38.3 ± 17.9 a	51.4 ± 7.1 a	50.0 ± 15.1 a	40.7 ± 11.8 a	0.092

Different letters within rows indicate significant differences (LSD, *p* < 0.05). “PA”: *Pediococcus acidilactici*, “SC”: *Saccharomyces cerevisiae*, “YP”: Yeast prebiotic.

## Data Availability

The data that support the findings of this study are available from the corresponding author upon reasonable request.
